# Association of spicy food consumption with colorectal polyp and adenoma prevalence: findings from the Lanxi Pre-Colorectal Cancer Cohort (LP3C)

**DOI:** 10.3389/fnut.2025.1642192

**Published:** 2025-09-09

**Authors:** Peiling Hu, Xinran Yan, Xiaodong Hu, Xunan Lin, Jing Zhao, Fuzhen Pan, Xiaohui Liu, Hao Ye, Pan Zhuang, Yu Zhang, Weifang Zheng, Jingjing Jiao

**Affiliations:** ^1^Lanxi Red Cross Hospital, Jinhua, Zhejiang, China; ^2^Department of Nutrition, School of Public Health, Zhejiang University School of Medicine, Hangzhou, Zhejiang, China; ^3^Lanxi Hospital of Traditional Chinese Medicine, Jinhua, Zhejiang, China; ^4^Zhejiang Key Laboratory for Agro-Food Processing, Department of Food Science and Nutrition, Fuli Institute of Food Science, College of Biosystems Engineering and Food Science, Zhejiang University, Hangzhou, Zhejiang, China

**Keywords:** colorectal polyps, adenoma, spicy food, Lanxi Pre-colorectal Cancer Cohort, diet

## Abstract

**Objective:**

This study examined the relationship between habitual spicy food intake and the risk of colorectal polyps and adenomas in a high-risk Chinese cohort.

**Methods:**

We analyzed baseline data from 14,907 participants aged 40–80 years enrolled in the Lanxi Precolorectal Cancer Cohort (LP3C) between March 2018 and December 2022. Dietary intake was assessed using a validated, single-administered baseline food frequency questionnaire (FFQ), with food intake frequency categorized into quartiles for analysis. Endoscopically confirmed colorectal lesions were histologically characterized. Multivariable-adjusted logistic regression models (adjusted for age, sex, body mass index (BMI), smoking, etc.) quantified lesion risks across spicy food consumption quartiles, with restricted cubic spline analyses evaluating non-linear exposure-response relationships.

**Results:**

Among 4,797 identified colorectal polyps and 2,607 adenomas, escalating spicy food intake exhibited a significant positive association with polyp risk (quartile 4 vs. quartile 1 OR: 1.24, 95% CI 1.13–1.37; P for trend < 0.001), contrasting with non-significant adenoma associations (quartile 4 vs. quartile 1 OR: 1.07, 95% CI 0.94–1.20; P for trend = 0.146), which was not clinically meaningful. Restricted cubic spline modeling revealed a non-linear relationship between spicy food intake and polyp risk (P for non-linearity < 0.001), characterized by initial risk elevation followed by a slight decrease with increasing consumption levels. Stratified analyses demonstrated consistent positive associations for polyp subgroups including small (≤5 mm) and large (>5 mm) lesions, single and multiple presentations, Yamada type classifications (≤II or ≥III), and both distal/proximal colonic locations (all P for trend ≤ 0.014).

**Conclusion:**

Our findings identify spicy food consumption as an independent dietary correlate of colorectal polyp formation in high-risk Chinese adults, with differential risk patterns across lesion subtypes and anatomical sites. These novel epidemiological findings suggest that limiting spicy food consumption may reduce polyp risk in populations at high risk of colorectal cancer.

## 1 Introduction

Global epidemiological surveillance data from the GLOBOCAN 2022 registry reveal colorectal cancer (CRC) accounted for 1.92 million incident cases and 900,000 mortality events worldwide in 2022, ranking as the second leading cause of cancer mortality globally and third most prevalent malignancy across both sexes (1). China’s evolving disease profile shows CRC persistently ranking among the top five malignancies for both incidence and mortality, with national surveillance data indicating an accelerating burden trajectory (1–4). The clinical significance of colorectal polyps and adenomas, well-established precursor lesions with malignant potential, is highlighted by longitudinal studies demonstrating substantially elevated 10-year CRC incidence rates among affected populations (5, 6). These epidemiological patterns emphasize the critical importance of targeted prevention strategies for precancerous colorectal lesions as a fundamental component of comprehensive CRC risk reduction initiatives.

Accumulating evidence suggests that dietary factors influence the risk of colorectal polyps and adenomas (7–12). Spicy cuisine - characterized by culinary preparations incorporating chili peppers or derived sauces - demonstrates substantial regional consumption variation within China, particularly in gastronomically distinct areas (13, 14). While capsaicin exhibits dual oncological properties through potential genotoxic effects and chemopreventive mechanisms, current evidence regarding its colorectal carcinogenicity remains inconclusive (15, 16). Contemporary investigations of spicy food’s role in colorectal carcinogenesis have yielded conflicting results, with the landmark China Kadoorie Biobank (CKB) cohort analysis identifying a modest inverse association between habitual consumption and rectal cancer risk (13, 15, 17–20). Recent case-control analyses comparing early-onset and late-onset colorectal cancer populations revealed a statistically significant elevation in spicy food exposure among younger patients, highlighting the need for focused investigation into dietary patterns preceding premalignant lesion development (18).

While current research presents divergent perspectives on the role of spicy foods in colorectal carcinogenesis, investigations into their association with CRC precursor lesions, particularly polyps and adenomas, remain lacking. Dietary factors exert heterogeneous effects across distinct polyp subtypes (21, 22). The associations between spicy food consumption and polyps categorized by anatomical location, Yamada classification, or multiplicity remain to be elucidated.

To address the above-mentioned knowledge gaps, we conducted the first large-scale epidemiological assessment of spicy food’s association with both colorectal polyps and adenomas within a high-risk Chinese population. Second, we employ restricted cubic spline (RCS) regression to characterize potential non-linear dose-response relationships between consumption level and polyp risk. Third, our stratified analysis of clinicopathological features leverages standardized endoscopic surveillance and histopathological verification to elucidate subtype-specific susceptibility patterns, advancing understanding of dietary interactions with early colorectal neoplasia development.

## 2 Materials and methods

### 2.1 Participants

The Lanxi Pre-Colorectal Cancer Cohort (LP3C) study was established in Zhejiang Province’s CRC high-incidence region to implement a 5-year population-based screening program targeting 500,000 residents aged 40–80 years with≥6 months’ residency in Jinhua. Between March 2018 and December 2022, we recruited high-risk individuals from 16 administrative units (6 subdistricts, 7 towns, 3 villages) meeting ≥1 of the following criteria: (1) personal history of colorectal polyps or CRC; (2) first-degree relative with CRC; (3) positive fecal immunochemical test (FIT) results (23). This study was approved by the ethical committee of Lanxi Red Cross Hospital (No. 20180302).

Trained interviewers administered standardized questionnaires capturing demographic, socioeconomic, dietary, and lifestyle characteristics, along with comprehensive medical histories. Participants underwent protocol-defined colonoscopy at Lanxi Red Cross Hospital within 30 days of enrollment. Full methodological details appear in our previous publication (24).

**FIGURE 1:**
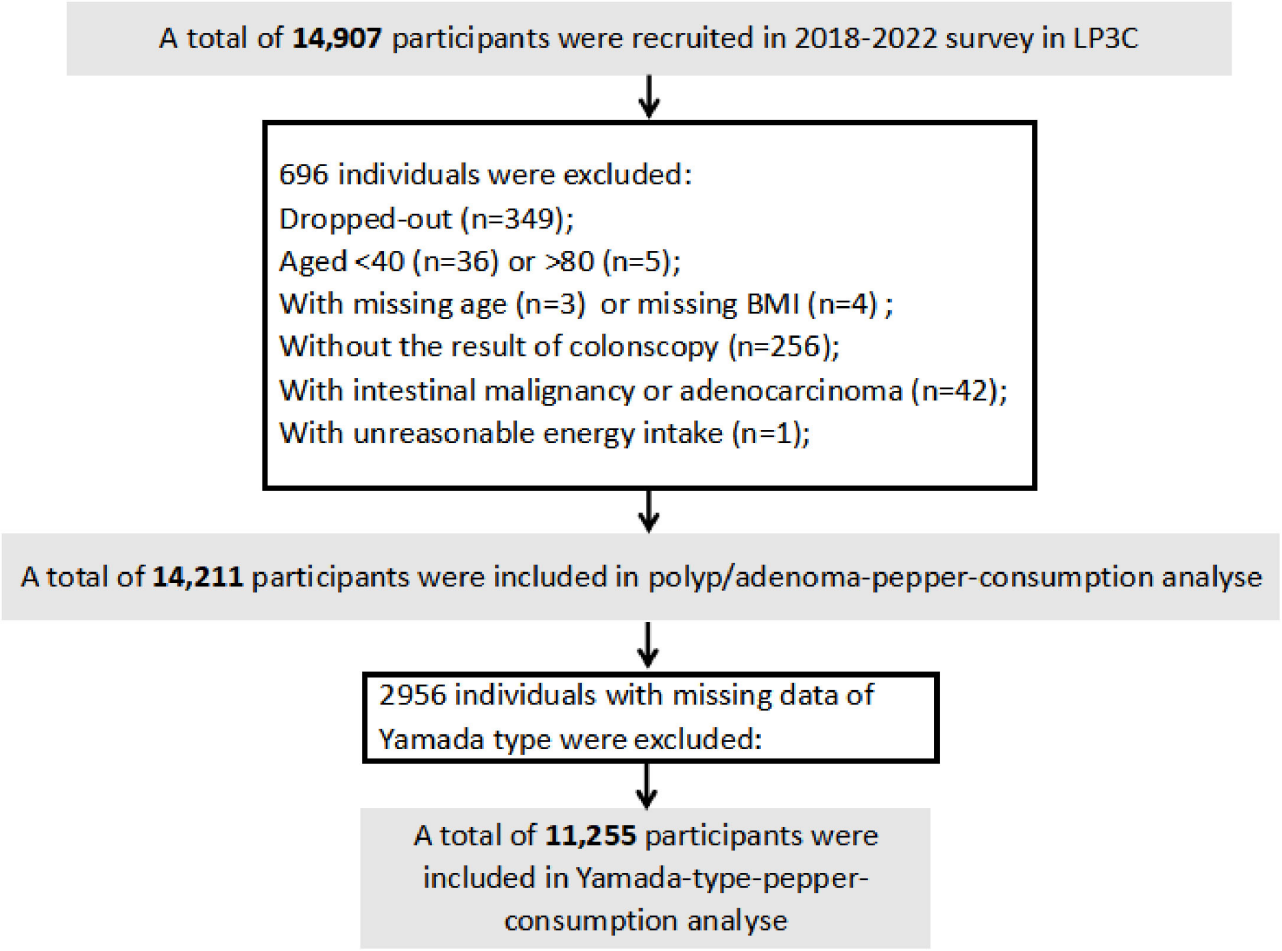
Flow chart of study participants in the current LP3C study.

From 14,907 initially enrolled participants, we excluded individuals who withdrew participation (*n* = 349), had age < 40/>80 years (*n* = 41), missing age/BMI data (*n* = 3/4), incomplete colonoscopy records (*n* = 256), confirmed intestinal malignancies (*n* = 42), or implausible energy intake values (*n* = 1), yielding 14,211 eligible subjects. For Yamada classification analyses, 11,255 participants remained after excluding 2,956 cases with missing endoscopic classification data (Figure 1).

### 2.2 Dietary assessment and covariates

Dietary intake was assessed using a validated FFQ adapted from the China Kadoorie Biobank FFQ (4, 25). Its reliability was further verified by two 24-h dietary recalls (24, 26, 27). Diet data from FFQ included 15 food categories, including rice, wheat, other staple foods (e.g., corn, millet, black rice, etc.), unprocessed red meat, processed red meat, poultry, fish or seafood, fresh eggs, fresh vegetables, soy products, preserved vegetables, fresh fruit, milk or fermented dairy products (e.g., cheese, yogurt, etc.), and spicy foods. Frequency of consumption was graded into five levels (never, daily, weekly, monthly, annual). Trained interviewers conducted questionnaires in person, using food model atlases and measurement tools (e.g., glasses, plates, bowls, spoons, etc.) to help participants estimate their portions. Food data were converted into intakes of dietary nutrients (fat, protein, fiber, and cholesterol) and total energy by The China Food Composition Tables (2018) (28). Additionally, a Healthy Diet Score was calculated based on ten major dietary components (24, 29), with favorable dietary factor (fish or seafood intake, fruit intake, vegetable intake, dairy product intake, wholegrain intake, tea intake) scored as 1 if intake was above the study population median (>median), and favorable dietary factor (processed red meat intake, unprocessed red meat intake, preserved vegetable intake and refined grain intake) scored as 1 if intake was at or below median (≤median), with higher scores indicating healthier eating on a scale of 0–10.

Other information, including demographic characteristics (age, gender, education level, annual household income), personal and family history of colorectal cancer, lifestyle factors (physical activity, smoking status, and alcohol consumption), and medication use (aspirin, vitamins, and calcium supplements), were collected and recorded by trained medical staff with detailed questionnaires. Body weight and height were measured using anthropometric methods, and BMI was calculated as weight (kg) divided by height (m) squared. According to Chinese standards, participants were classified as underweight (<18.5 kg/m^2^), normal weight (18.5–23.9 kg/m^2^), overweight (24–27.9 kg/m^2^), and obese (≥28 kg/m^2^) (29–31). Each participant’s weekly physical activity level was calculated in metabolic equivalent task hours (MET-h/wk) by Compendium of Physical Activities (32).

### 2.3 Assessment of spicy food consumption

Similar to the definition of spicy food in China Kadoorie Biobank (13, 33), spicy food intake is defined as: (1) direct consumption of fresh chili peppers; (2) addition of fresh/dried chili peppers, chili oil/sauce/paste, curry or other “hot” spices during cooking; or (3) incorporation of chili oil/sauce/paste into prepared dishes during meals. Spicy food consumption frequency at baseline was categorized as: never, less than 1 day per week, 1–2 days per week, 3–5 days per week, or 6–7 days per week. Those who did not choose any of the options were considered to have never consumed spicy food.

### 2.4 Outcome ascertainment

Qualified proctologists conducted the electronic colonoscopy procedures. In cases where colorectal polyps measuring ≥5 mm in diameter were detected during the examination, the polyps were generally removed unless the patients strongly opposed, were on anticoagulant therapy (e.g., aspirin), or had inadequate bowel preparation. The removed polyps were immediately sent to the Pathology Department for histopathological examination. In terms of anatomic subsite, polyps found in the cecum, ascending colon, hepatic flexure, transverse colon, or splenic flexure were categorized as proximal, those in the descending or sigmoid colon as distal, and those in the rectum or rectosigmoid junction as rectal. Participants harboring tubular adenomas, villous adenomas, or tubulovillous adenomas are classified as adenoma carriers. Additionally, colorectal polyps were graded by size into small (diameter < 10 mm) and large (diameter ≥ 10 mm) categories (5, 29); by multiplicity into solitary (quantity = 1) and multiple (quantity > 1); as well as by Yamada type, being either ≤II (been diagnosed with Yamada I or II) or ≥III (been diagnosed with Yamada III or IV at least once) (34).

### 2.5 Statistical analysis

The intake of foods is expressed in terms of energy density (g 2000 kcal^–1^⋅d^–1^) using the nutrient density method (29, 35). Continuous variables were presented as means ± standard errors, and categorical variables were denoted by numeric values accompanied by percentages (%) in the description of baseline characteristics.

The association between spicy food consumption and the prevalence of colorectal polyps and adenoma was assessed using multivariate logistic regression analysis, which derived ORs and 95% CIs. Spicy food intake was categorized into quartiles (Q1: ≤25th percentile; Q2: 25th–50th; Q3: 50th–75th; Q4: >75th percentile) based on the population distribution. Incorporating known risk factors as covariates within the models, a stepwise regression approach was employed. Model 1 was age- and sex-adjusted. Model 2 incorporated additional adjustments for BMI (<18.5, 18.5–24, 24–28, >28, in kg/m2), smoking (never, past smokers with <25 pack-years or ≥25 pack-years, current smokers with <25 pack-years or ≥25 pack-years), alcohol consumption (never, ≤25 mL for men and ≤15 mL for women, >25 mL for men and >15 mL for women), household annual income (Yuan), physical activity (MET-h/wk), vitamin supplement use (yes or no), family colorectal cancer history (yes or no), regular aspirin use (yes or no) and educational level (<middle school or ≥middle school). Building on model 2, model 3 further adjusted for total energy intake (quartile), intake of fresh vegetable (quartile), intake of fruit vegetable (quartile) and intake of unprocessed red meat (quartile), while model 4 further adjusted for total energy intake (quartile) and healthy diet score (quartile). The median of the respective category was treated as a continuous variable for linear trends.

In secondary analysis, we calculated the ORs and 95%CIs of polyps and adenomas with varying histopathological features, including size, multiplicity and anatomic location. Restricted cubic splines were used to explore potential non-linear relationships between spicy food consumption and colorectal polyp risk. Sensitivity analyses were conducted for assessment of the model robustness. We respectively excluded individuals with extreme energy intake (<800 kcal/day or >4200 kcal/day for males and<600 kcal/day or >3500 kcal/day for females), those with extreme BMI values (<15 or >40 kg/m^2^), adjusted for calcium supplement intake, and baseline diabetes and baseline cancer status to determine whether the previously observed associations persisted. In the subgroup analysis, we further assessed associations in subgroups stratified based on the following covariates: age (<60 or ≥60 years), BMI (<24 or ≥24 kg/m^2^), sex (males or females), physical activity (<median or ≥median), smoking status (non-smokers or former/current smokers), alcohol intake (non-drinkers or drinkers), education (<middle school or ≥middle school), vitamin consumption (<median or ≥median), family colon cancer history (yes or no), Aspirin use (yes or no), total energy intake (<median or ≥ median) and healthy diet score (<median or ≥median).

All analyses were performed using SAS 9.4 (SAS Institute), with restricted cubic spline (RCS) modeling implemented through R statistical software (version 4.4.3) employing the rms package (v7.0-0). Statistical significance was defined using a two-tailed α-level of 0.05, with effect estimates expressed as odds ratios (ORs) accompanied by 95% confidence intervals (CIs).

## 3 Results

### 3.1 Characteristics

During the March 2018-December 2022 observation period, the analytical cohort comprising 14,211 participants revealed 4,797 colorectal polyp cases and 2,607 adenoma diagnoses. At baseline, individuals who consume more spicy food tend to be male (Q4: 57.44% vs. Q1: 46.74%) and have higher household incomes (highest level, Q4: 25.59% vs. Q1: 18.82%). In addition, they were more likely to have a higher BMI (highest level, Q4: 9.07% vs. Q1: 6.75%) and were more likely to smoke (highest level, Q4: 17.45% vs. Q1: 8.91%) and drink alcohol (highest level, Q4: 49.44% vs. Q1: 24.00%). In terms of diet, they consumed fewer dairy products (Q4: 31.80 ± 73.68 g 2000 kcal^–1^⋅d^–1^ vs. Q1: 39.34 ± 84.65 g 2000 kcal^–1^⋅d^–1^) and fresh vegetables (Q4: 212.17 ± 104.82 g 2000 kcal^–1^⋅d^–1^ vs. Q1: 233.77 ± 114.94 g 2000 kcal^–1^⋅d^–1^), while the intake of preserved vegetables (Q4: 7.32 ± 15.89 g 2000 kcal^–1^ d^–1^ vs. Q1: 3.77 ± 10.87 g 2000 kcal^–1^⋅d^–1^), tea (Q4: 646.88 ± 1535.03 g 2000 kcal^–1^⋅d^–1^ vs. Q1: 313.45 ± 961.19 g 2000 kcal^–1^⋅d^–1^), seafood (Q4: 19.71 ± 27.15 g 2000 kcal^–1^⋅d^–1^ vs. Q1: 13.20 ± 21.48 g 2000 kcal^–1^⋅d^–1^) and poultry (Q4: 6.86 ± 13.30 g 2000 kcal^–1^⋅d^–1^ vs. Q1: 4.36 ± 8.51 g 2000 kcal^–1^⋅d^–1^) was higher (Table 1).

**TABLE 1:** Baseline characteristics of study participants according to quartiles of spicy food consumption^a^.

Characteristics	Quartiles of spicy food consumption (g 2000 kcal^–1^⋅d^–1^)			
	Q1^b^	Q2	Q3	Q4
*N*	4,771	2,412	3,292	3,736
Range (g 2000 kcal^–1^⋅d^–1^)	0	0∼2.9	2.9∼16.4	≥16.4
Age (yr)	61.40 ± 7.68	61.27 ± 8.01	60.02 ± 7.98	59.15 ± 7.83
**Sex, *n* (%)**				
Male	2,230 (46.74)	1,225 (50.79)	1,795 (54.53)	2,146 (57.44)
Female	2,541 (53.26)	1,187 (49.21)	1,497 (45.47)	1,590 (42.56)
**Family history of colorectal cancer, *n* (%)**				
No	4,330 (90.76)	2,243 (92.99)	2,995 (90.98)	3,378 (90.42)
Yes	313 (6.56)	129 (5.35)	202 (6.14)	217 (5.81)
**Body mass index (kg/m^2^)**				
<18.5, *n* (%)	241 (5.05)	65 (2.69)	89 (2.70)	106 (2.84)
18.5–24.0, *n* (%)	2,682 (56.21)	1,286 (53.32)	1,704 (51.76)	1,887 (50.51)
24.0–28, *n* (%)	1,526 (31.98)	893 (37.02)	1,219 (37.03)	1,404 (37.58)
>28, *n* (%)	322 (6.75)	168 (6.97)	280 (8.51)	339 (9.07)
**Educational level**				
<Middle school, *n* (%)	4,154 (87.07)	2,050 (84.99)	2,771 (84.17)	3,183 (85.20)
≥Middle school, *n* (%)	617 (12.93)	362 (15.01)	521 (15.83)	553 (14.80)
**Household income (Yuan/yr), *n* (%)**				
< 30,000	1,771 (37.12)	870 (36.07)	1,002 (30.44)	1,079 (28.88)
30,000–100,000	2,008 (42.09)	1,000 (41.46)	1,427 (43.35)	1,624 (43.47)
> 100,000	898 (18.82)	530 (21.97)	791 (24.03)	956 (25.59)
**Physical activity (MET-h/wk^c^)**				
Q1 (<75.0), *n* (%)	1,228 (25.74)	662 (27.45)	839 (25.49)	841 (22.51)
Q2 (75.3∼139.5), *n* (%)	1,287 (26.98)	617 (25.58)	830 (25.21)	808 (21.63)
Q3 (140.0∼253.8), *n* (%)	1,147 (24.04)	622 (25.79)	806 (24.48)	895 (23.96)
Q4 (≥254.0), *n* (%)	1,109 (23.24)	511 (21.19)	817 (24.82)	1,192 (31.91)
**Smoke, *n* (%)**				
Never	3,358 (70.38)	1,510 (62.60)	2,052 (62.33)	2,160 (57.82)
**Past smokers**				
<25 pack-years	383 (8.03)	229 (9.49)	253 (7.69)	302 (8.08)
≥25pack-years	307 (6.43)	179 (7.42)	207 (6.29)	232 (6.21)
**Current smokers**				
<25 pack-years	298 (6.25)	186 (7.71)	295 (8.96)	390 (10.44)
≥25pack-years	425 (8.91)	308 (12.77)	485 (14.73)	652 (17.45)
**Alcohol drinker, *n* (%)**				
Never	3,331 (69.82)	1,486 (61.61)	1,726 (52.43)	1,631 (43.66)
≤25 ml/d for men, ≤15 ml/d for women	295 (6.18)	178 (7.38)	257 (7.81)	258 (6.91)
>25 ml/d for men, >15 ml/d for women	1,145 (24.00)	748 (31.01)	1,309 (39.76)	1,847 (49.44)
**Vitamin supplement intake, *n* (%)**				
No	4,716 (98.85)	2,375 (98.47)	3,226 (98.00)	3,691 (98.80)
Yes	55 (1.15)	37 (1.53)	66 (2.00)	45 (1.20)
**Calcium supplement intake, *n* (%)**				
No	4,303 (90.19)	2,040 (84.58)	2,922 (88.76)	3,405 (91.14)
Yes	468 (9.81)	372 (15.42)	370 (11.24)	331 (8.86)
**Regular aspirin use, *n* (%)**				
No	4,679 (98.07)	2,344 (97.18)	3,251 (98.75)	3,685 (98.63)
Yes	91 (1.91)	68 (2.82)	41 (1.25)	49 (1.31)
Cancer, *n* (%)	114 (2.39)	47 (1.95)	44 (1.34)	18 (0.48)
**Dietary intake**				
Total energy (kcal⋅d^–1^)	1,980.26 ± 682.19	2,158.97 ± 769.98	2,175.59 ± 770.27	2,295.18 ± 850.46
Whole grains (g 2000 kcal^–1^⋅d^–1^)	412.33 ± 136.86	454.54 ± 146.61	431.40 ± 138.35	429.33 ± 140.20
Refined grains (g 2000 kcal^–1^⋅d^–1^)	405.82 ± 137.05	445.84 ± 147.35	425.30 ± 138.08	423.81 ± 140.09
Total dairy products (g 2000 kcal^–1^⋅d^–1^)	39.34 ± 84.65	35.00 ± 76.09	32.66 ± 67.91	31.80 ± 73.68
Fresh vegetables (g 2000 kcal^–1^⋅d^–1^)	233.77 ± 114.94	221.80 ± 111.13	219.98 ± 105.91	212.17 ± 104.82
Preserved vegetables (g 2000 kcal^–1^⋅d^–1^)	3.77 ± 10.87	5.11 ± 10.28	5.14 ± 11.31	7.32 ± 15.89
Fruits (g 2000 kcal^–1^⋅d^–1^)	101.81 ± 101.24	84.33 ± 92.56	92.37 ± 93.09	90.47 ± 112.55
Tea (g 2000 kcal^–1^⋅d^–1^)	313.45 ± 961.19	361.75 ± 912.67	445.24 ± 1104.47	646.88 ± 1535.03
Seafood (g 2000 kcal^–1^ d^–1^)	13.20 ± 21.48	14.17 ± 20.19	18.64 ± 24.34	19.71 ± 27.15
Poultry (g 2000 kcal^–1^ d^–1^)	4.36 ± 8.51	4.74 ± 8.16	6.26 ± 10.53	6.86 ± 13.30
Processed red meat (g 2000 kcal^–1^⋅ d^–1^)	0.45 ± 1.68	0.62 ± 1.82	0.84 ± 4.37	0.98 ± 3.17
Unprocessed red meat (g 2000 kcal^–1^⋅ d^–1^)	49.06 ± 51.61	31.39 ± 32.83	47.02 ± 43.36	53.64 ± 48.25
Egg (g 2000 kcal^–1^⋅d^–1^)	31.03 ± 29.42	31.86 ± 30.35	30.53 ± 27.88	27.20 ± 27.39
Healthy diet score	4.99 ± 1.62	4.73 ± 1.66	4.81 ± 1.69	4.58 ± 1.62

^a^Data are percentages or means (standard errors) unless indicated otherwise.

^b^Q, quartile. Q1: ≤25th percentile; Q2: 25th–50th; Q3: 50th–75th; Q4: >75th percentile.

^c^MET-h/wk: metabolic equivalent task hours per week.

### 3.2 Spicy food consumption and colorectal polyp and adenoma prevalence

In age- and sex- adjusted model 1, there was a significant dose-response positive association between spicy food consumption and colorectal polyps [OR_Q2 vs. Q1_ (95% CI): 1.18 (1.06–1.32); OR_Q3 vs. Q1_ (95% CI): 1.29 (1.17–1.43); OR_Q4 vs. Q1_ (95% CI): 1.24 (1.13–1.37); *P-trend* < 0.001], this association remained significant after further adjustment for demographic characteristics in model 2 [OR_Q2 vs. Q1_ (95% CI): 1.11 (0.99–1.24); OR_Q3 vs. Q1_ (95% CI): 1.21 (1.09–1.33); OR_Q4 vs. Q1_ (95% CI): 1.13 (1.02–1.25); *P-trend* = 0.004]. Subsequently, the positive association between spicy food consumption and colorectal polyps persisted in model 3 [OR_Q2 vs. Q1_ (95% CI): 1.08 (0.97–1.21); OR_Q3 vs. Q1_ (95% CI): 1.20 (1.08–1.32); OR_Q4 vs. Q1_ (95% CI): 1.12 (1.01–1.24); *P-trend* = 0.006] and 4 [OR _rm Q2 vs. Q1_ (95% CI): 1.10 (0.98–1.22); OR_Q3 vs. Q1_ (95% CI): 1.20 (1.08–1.32); OR_Q4 vs. Q1_ (95% CI): 1.12 (1.01–1.23); *P-trend* = 0.007], which were developed based on model 2, with adjustments for energy and dietary intake in model 3. Adjusting for energy and healthy diet score in model 4 based on model 2 also yielded similar positive associations (Table 2).

**TABLE 2:** Multivariable-adjusted ORs (95% CIs) of spicy food consumption with the prevalence of colorectal polyps or adenomas^a^.

	Quartiles of spicy food consumption (g⋅2000 kcal^–1^⋅d^–1^)				*P*-trend
	Q1^a^	Q2^b^	Q3^c^	Q4^d^	
Range (g⋅2000 kcal^–1^⋅d^–1^)	0	0∼2.9	2.9∼16.4	≥16.4	
**Polyp**					
Cases/*n*	1,465/4,771	839/2,412	1,189/3,292	1,304/3,736	
Model 1^e^	1 (Ref.)	1.18 (1.06–1.32)	1.29 (1.17–1.43)	1.24 (1.13–1.37)	<0.001
Model 2^f^	1 (Ref.)	1.11 (0.99–1.24)	1.21 (1.09–1.33)	1.13 (1.02–1.25)	0.004
Model 3^g^	1 (Ref.)	1.08 (0.97–1.21)	1.20 (1.08–1.32)	1.12 (1.01–1.24)	0.006
Model 4^h^	1 (Ref.)	1.10 (0.98–1.22)	1.20 (1.08–1.32)	1.12 (1.01–1.23)	0.007
**Adenoma**					
Cases/*n*	764/4,771	493/2,412	675/3,292	675/3,736	
Model 1	1 (Ref.)	1.33 (1.17–1.51)	1.37 (1.22–1.54)	1.18 (1.05–1.33)	0.001
Model 2	1 (Ref.)	1.26 (1.11–1.44)	1.28 (1.14–1.45)	1.08 (0.96–1.22)	0.085
Model 3	1 (Ref.)	1.22 (1.07–1.39)	1.27 (1.13–1.43)	1.07 (0.95–1.21)	0.101
Model 4	1 (Ref.)	1.24 (1.09–1.42)	1.27 (1.13–1.43)	1.07 (0.94–1.20)	0.146

MET-h/wk, metabolic equivalent task hours per week; BMI, body mass index; n, sample size; Q, quartile.

^a^Q1: ≤25th percentile.

^b^Q2: 25th–50th.

^c^Q3: 50th–75th.

^d^Q4: >75th percentile.

^e^Model 1 was adjusted for age and sex.

^f^Model 2 was adjusted for model 1 plus BMI (<18.5, 18.5–24, 24–28, >28, in kg/m^2^), smoking (never, past smokers with <25 pack-years or ≥25 pack-years, current smokers with <25 pack-years or ≥25 pack-years), alcohol consumption (never, ≤25 mL for men and ≤15 mL for women, >25 mL for men and >15 mL for women), household annual income (Yuan), physical activity (MET-h/wk), vitamin supplement use (yes or no), history of family colorectal cancer (yes or no), regular aspirin use (yes or no), educational level (<middle school or ≥middle school).

^g^Model 3 was adjusted for model 2 plus total energy intake (quartile), intake of fresh vegetable (quartile), intake of fruit vegetable (quartile), intake of unprocessed red meat (quartile).

^h^Model 4 was adjusted for model 2 plus total energy intake (quartile), healthy diet score (quartile).

In addition, further analysis of colorectal polyp subtypes, including size, multiplicity, Yamada classification, and anatomical location, revealed that higher spicy food intake was strongly associated with larger polyp size (*P-trend* = 0.014), single (*P-trend* = 0.011) or multiple polyps (*P-trend* = 0.012), Yamada type II or less (*P-trend* = 0.002), and positively correlated with polyps located in both the distal (*P-trend* = 0.012) and proximal colon (*P-trend* = 0.004) (Table 3).

**TABLE 3:** Multivariable-adjusted ORs (95% CIs) of spicy food consumption with the prevalence of polyps according to subtypes^a^.

Subtypes		Quartiles of spicy food consumption (g⋅2000 kcal^−1^⋅d^−1^)				*P*-trend
		Q1^a^	Q2^b^	Q3^c^	Q4^d^	
Range (g⋅2000 kcal^−1^⋅d^−1^)		0	0∼2.9	2.9∼16.4	≥16.4	
**Size**						
<10 mm	Cases	930	598	777	726	
	OR (95% CI)	1 (Ref.)	0.99 (0.87–1.12)	1.15 (1.02–1.29)	1.14 (1.00–1.29)	0.009
≥10 mm	Cases	238	221	239	217	
	OR (95% CI)	1 (Ref.)	1.38 (1.13–1.69)	1.34 (1.10–1.64)	1.30 (1.06–1.59)	0.014
**Multiplicity**						
Single	Cases	829	513	679	626	
	OR (95% CI)	1 (Ref.)	0.98 (0.86–1.11)	1.15 (1.02–1.30)	1.13 (1.00–1.29)	0.011
Multiple	Cases	339	306	337	317	
	OR (95% CI)	1 (Ref.)	1.29 (1.08–1.54)	1.28 (1.08–1.52)	1.26 (1.05–1.51)	0.012
**Yamada type**						
≤II	Cases	717	515	643	596	
	OR (95% CI)	1 (Ref.)	1.09 (0.96–1.25)	1.22 (1.07–1.39)	1.20 (1.05–1.37)	0.002
≥III	Cases	451	304	373	347	
	OR (95% CI)	1 (Ref.)	1.02 (0.87–1.20)	1.13 (0.96–1.32)	1.12 (0.95–1.31)	0.109
**Anatomic Subsite**						
Distal colon	Cases	583	458	554	494	
	OR (95% CI)	1 (Ref.)	1.16 (1.00–1.34)	1.26 (1.09–1.44)	1.17 (1.01–1.35)	0.012
Proximal colon	Cases	506	392	458	424	
	OR (95% CI)	1 (Ref.)	1.18 (1.01–1.37)	1.24 (1.07–1.44)	1.23 (1.05–1.43)	0.004
Rectum	Cases	243	165	194	161	
	OR (95% CI)	1 (Ref.)	1.01 (0.82–1.25)	1.07 (0.87–1.31)	0.95 (0.76–1.18)	0.854

BMI, body mass index; MET-h/wk, metabolic equivalent task hours per week; Q, quartile.

*^a^*Q1: ≤25th percentile.

*^b^*Q2: 25th–50th.

*^c^*Q3: 50th–75th.

*^d^*Q4: >75th percentile. Model was adjusted for age, sex, BMI (<18.5, 18.5–24, 24–28, >28, in kg/m2), smoking (never, past smokers with <25 pack-years or ≥25 pack-years, current smokers with <25 pack-years or ≥25 pack-years), alcohol consumption (never, ≤25 mL for men and ≤15 mL for women, >25 mL for men and >15 mL for women), household annual income (Yuan), physical activity (MET-h/wk), vitamin supplement use (yes or no), history of family colorectal cancer (yes or no), regular aspirin use (yes or no), educational level (<middle school or ≥middle school), total energy intake (quartile), healthy diet score (quartile).

The association was only significant in model 1 when colorectal adenomas were analyzed as the outcome. Using the lowest quartile of spicy food intake as the reference, the ORs with 95%CIs for the highest quartile were 1.18 (1.05–1.33) in model 1 (*P-trend* = 0.001), 1.08 (0.96–1.22) in model 2 (*P-trend* = 0.085), 1.07 (0.95–1.21) in model 3 (*P-trend* = 0.101), and 1.07 (0.94–1.20) in model 4 (*P-trend* = 0.146) (Table 2).

### 3.3 RCS for spicy food consumption in relation to polyp risk

**FIGURE 2:**
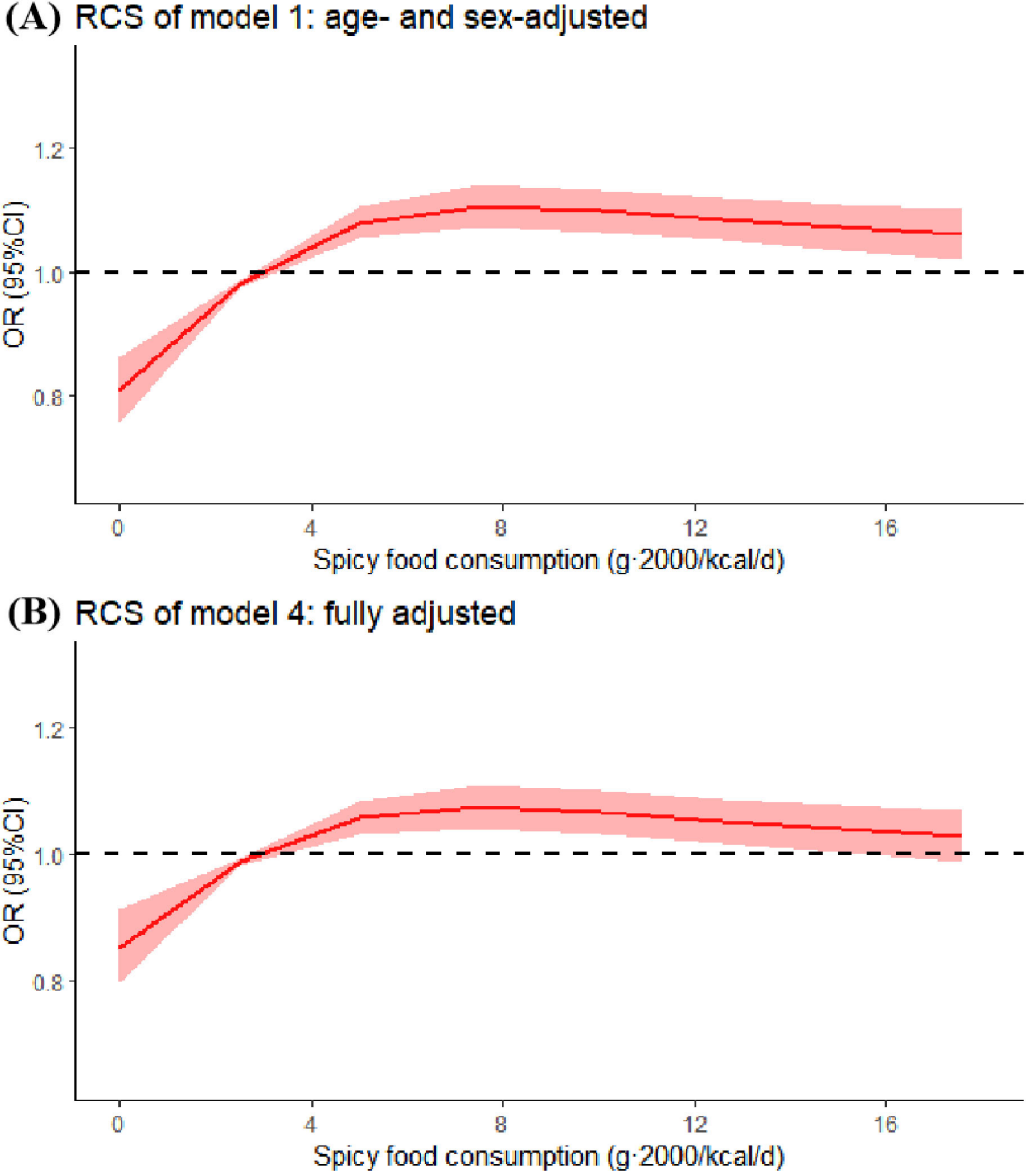
Restricted cubic spline (RCS) for spicy food consumption in model 1 (age- and sex-adjusted) and model 4 (fully adjusted). Odds ratios were estimated by restricted-cubic-spline regression adjusted for covariates in model 1 or 4. Shaded areas represent 95% confidence intervals.

The results of RCS analysis showed that there was a non-linear positive relationship between spicy food intake and risk of colorectal polyps in both model 1 (*P-non-liear* < 0.001) and model 4 (*P-non-liear* < 0.001). In Model 4, with increasing consumption of spicy foods, polyp risk first increased sharply and then decreased slightly (Figure 2).

### 3.4 Subgroup analyses and sensitivity analyses

In the subgroup analysis, when stratified by baseline age, BMI, sex, physical activity, smoking status, alcohol intake, education, vitamin consumption, family colon cancer history, aspirin use, total energy intake and healthy diet score, significant positive association between spicy food intake and the risk of colorectal polyps was observed among individuals with low physical activity levels, non-drinkers, and those without a family history of colorectal cancer. While there were no significant interactions observed in the analyses of adenomas (Supplementary Table 1).

To assess the reliability of the findings, we conducted sensitivity analyses by excluding individuals with extreme energy intake and extreme body mass index (BMI), and further adjusting for calcium supplement use and baseline diabetes status. The positive association between spicy food intake and colorectal polyps, as well as the lack of association with adenomas, remained unchanged, confirming the robustness of our findings (Supplementary Table 2).

## 4 Discussion

In this study, we revealed a significant positive relationship between spicy food intake and the risk of colorectal polyps, though no such association was observed for adenomas. Furthermore, spicy food intake was positively associated with polyp size across various subtypes, with notable trends observed for both small and large polyps. Similar associations were identified for multiple polyps, single polyps, polyps Yamade-typed less than II, and polyps located in both the distal and proximal colon.

Our study pioneers the quantification of spicy food intake in association with colorectal polyp risk in a Chinese population. There have been few studies on the association between spicy food intake and CRC. Some epidemiological studies support an adverse association between spicy food consumption and colorectal cancer risk, aligning with the key observations in our investigation (13, 15, 18). Studies have explored differences in dietary factors between early-onset and old-onset CRC, finding that early-onset CRC patients have higher spicy food intake (18). Meanwhile, a study looking at associations between various dietary types, foods or nutrients and colorectal cancer risk in Asian populations found a positive association between spicy food and CRC (15). This is consistent with our finding that spicy food intake is positively associated with the risk of colorectal polyps. However, studies have also found no statistically significant association between spicy food intake and CRC risk (13, 17, 19, 20). Researchers who studied chili peppers and CRC found that chili peppers did not increase or decrease CRC, which is consistent with our finding that spicy food intake is not significantly associated with colorectal adenoma (19). An investigation on spicy food consumption and gastrointestinal cancer risk in the CKB study identified a marginally inverse association with rectal cancer, but no significant association with colon cancer, they suggested that inverse association may be partly due to reverse causation (13). Unlike studies among China general population, our study population was at high risk for colorectal cancer, which might be susceptible to the potential pro-carcinogenic effects of capsaicin.

The association observed in RCS analysis in our study may reflect the dual role of bioactive compounds in spicy foods. At lower intake levels, pro-carcinogenic effects of capsaicin dominate, while higher doses may activate its protective pathways (15, 16, 20, 36–38). This hypothesis is supported by *in vitro* studies showing dose-dependent effects of capsaicin (38). Capsaicin is a pungent capsaicinoid compound produced as a secondary metabolite by pepper fruit (16). Several research findings indicate that low-dose capsaicin intake may contribute to the development of one or more types of gastrointestinal cancers (15, 36, 38). Diet intake of capsaicin caused duodenal tumors in Swiss albino mice (37). The potential mechanism of action may involve modulation of reactive oxygen species (ROS) generation, which activates the Akt/mTOR and STAT-3 signaling pathways in SW480 cells, upregulates MMP-2 and MMP-9 expression, induces epithelial-mesenchymal transition (EMT), ultimately promoting the development of CRC (38). At higher intake levels, capsaicin exhibits anti-cancer properties (16, 39–43). Research has demonstrated that higher doses of capsaicin may confer protective effects against gastrointestinal disorders through either inhibiting Helicobacter pylori growth or modulating interleukin expression (39, 40, 44).

Polyp size and shape strongly influence clinical outcomes (5, 34, 45). In this study, spicy food consumption was strongly correlated with polyp size, single or multiple, Yamada type II or less polyps, and positively correlated with anatomical location of polyps occurring in the distal and proximal colon, indicating severity and prevalence. The observed link between spicy food consumption and rectal polyps may arise from distinct functional roles and disease mechanisms in the colon versus the rectum, despite their anatomical similarities and close connection (46).

This investigation demonstrates unique value in the following aspects. First, it is the first study to quantify spicy food intake and its association with polyps and adenomas in a high-risk population. Second, we have subtyped colorectal polyps, which enabled more precise localization of the effects of spicy food consumption. Furthermore, building upon previous research, we analyzed the pre-colorectal cancer population and conducted separate analyses for polyps and adenomas, which are precancerous lesions with significant preventive implications, thereby identifying potential predictors of colorectal cancer progression.

On the other hand, this study has several limitations. First, inevitable measurement errors in self-reported intake may attenuate the true association between spicy food consumption and colorectal polyp risk. Second, the findings may have limited generalizability due to the unique dietary patterns and lifestyle habits specific to the Chinese population. Although we did not account for genetic factors through genotyping, the model maintained robust stability after excluding participants with a family history of colorectal cancer and a personal history of the disease. Third, we did not consider the relative contribution of active compounds across diverse chili products, warranting more refined categorization in future studies. Finally, as an observational study, this analysis cannot establish causal relationships, and we acknowledge the potential presence of uncontrolled or unmeasured confounding factors.

## 5 Conclusion

Collectively, our research demonstrates a relationship between higher spicy food intake and elevated colorectal polyp risk within high-risk populations in China. Our results suggest that spicy food intake should be minimized for people at high risk of CRC to prevent colorectal polyps.

## Data Availability

The raw data supporting the conclusions of this article will be made available by the authors, without undue reservation.
