# Supplementary material for: Association of spicy food consumption with colorectal polyp and adenoma prevalence: findings from the Lanxi Pre-Colorectal Cancer Cohort (LP3C)
Source: Front Nutr. 2025 Sep 9;12:1642192. doi: 10.3389/fnut.2025.1642192 (PMC12454312; doi:10.3389/fnut.2025.1642192)
Supplement: Supplementary file 1 [file Table_1.docx]

Supplementary Material

# Supplementary Tables

## Table of Contents

**Supplementary Table 1.** Subgroup analyses for multivariable-adjusted ORs (95% CIs) of spicy food consumption with the prevalence of polyps or adenomas

**Supplementary Table 2.** Sensitivity analyses for the multivariable-adjusted ORs (95% CIs) of colorectal polyp or adenoma prevalence according to spicy food consumption

## Supplementary Tables

| **Supplement Table 1.** Subgroup analyses for multivariable-adjusted ORs (95% CIs) of spicy food consumption with the prevalence of polyps or adenomas ^a^. | | | | | | | |
| --- | --- | --- | --- | --- | --- | --- | --- |
|  | Cases/n | Quartiles of spicy food consumption (g·2000 kcal^-1^·d^-1^) | | | | P-trend | P for interaction |
|  |  | Q1 | Q2 | Q3 | Q4 |  |  |
| **Polyp** |  |  |  |  |  |  |  |
| Age |  |  |  |  |  |  | 0.368 |
| <60 year | 1,778/6,745 | 1 (Ref.) | 1.24 (1.04-1.48) | 1.26 (1.08-1.48) | 1.06 (0.91-1.23) | 0.440 |  |
| ≥60 year | 3,019/7,466 | 1 (Ref.) | 1.03 (0.90-1.19) | 1.16 (1.02-1.32) | 1.18 (1.03-1.34) | 0.004 |  |
| BMI |  |  |  |  |  |  | 0.438 |
| <24 kg/m^2^ | 2,543/8,060 | 1 (Ref.) | 1.20 (1.04-1.39) | 1.33 (1.16-1.52) | 1.14 (1.00-1.31) | 0.008 |  |
| ≥24 kg/m^2^ | 2,254/6,151 | 1 (Ref.) | 0.97 (0.82-1.14) | 1.04 (0.90-1.21) | 1.06 (0.91-1.23) | 0.353 |  |
| Sex |  |  |  |  |  |  | 0.730 |
| Men | 3,173/7,396 | 1 (Ref.) | 1.00 (0.87-1.16) | 1.15 (1.01-1.31) | 1.07 (0.94-1.21) | 0.152 |  |
| Women | 1,624/6,815 | 1 (Ref.) | 1.22 (1.04-1.44) | 1.25 (1.08-1.46) | 1.18 (1.01-1.38) | 0.014 |  |
| Physical activity |  |  |  |  |  |  | 0.041 |
| <Median | 2,574/7,112 | 1 (Ref.) | 1.19 (1.02-1.37) | 1.27 (1.11-1.46) | 1.19 (1.04-1.38) | 0.003 |  |
| ≥Median | 2,223/7,099 | 1 (Ref.) | 1.00 (0.85-1.18) | 1.12 (0.97-1.30) | 1.04 (0.90-1.20) | 0.373 |  |
| Smoking |  |  |  |  |  |  | 0.094 |
| Nonsmoker | 2,415/9,080 | 1 (Ref.) | 1.11 (0.96-1.28) | 1.25 (1.10-1.42) | 1.23 (1.08-1.40) | <0.001 |  |
| Former/current smoker | 2,382/5,131 | 1 (Ref.) | 1.07 (0.90-1.27) | 1.12 (0.95-1.31) | 0.98 (0.84-1.15) | 0.934 |  |
| Alcohol consumption |  |  |  |  |  |  | 0.008 |
| Nondrinker | 2,400/8,174 | 1 (Ref.) | 1.19 (1.03-1.36) | 1.23 (1.08-1.40) | 1.27 (1.11-1.46) | <0.001 |  |
| Drinker | 2,397/6,037 | 1 (Ref.) | 0.95 (0.80-1.14) | 1.13 (0.96-1.31) | 0.97 (0.84-1.12) | 0.990 |  |
| Educational level |  |  |  |  |  |  | 0.703 |
| < middle school | 4,100/12,158 | 1 (Ref.) | 1.13 (1.01-1.27) | 1.23 (1.11-1.37) | 1.13 (1.02-1.26) | 0.005 |  |
| ≥ middle school | 697/2,053 | 1 (Ref.) | 0.88 (0.65-1.19) | 0.97 (0.74-1.27) | 1.02 (0.78-1.34) | 0.804 |  |
| Vitamin consumption |  |  |  |  |  |  | 0.533 |
| <Median | 4,747/14,008 | 1 (Ref.) | 1.05 (1.01-1.08) | 0.96 (0.86-1.07) | 1.08 (0.96-1.21) | 0.006 |  |
| ≥Median | 50/203 | 1 (Ref.) | 0.67 (0.41-1.07) | 1.26 (0.83-1.91) | 1.03 (0.65-1.62) | 0.835 |  |
| Family coloncancer history |  |  |  |  |  |  | 0.025 |
| No | 4,390/12,946 | 1 (Ref.) | 1.06 (1.02-1.09) | 0.95 (0.84-1.06) | 1.09 (0.97-1.23) | 0.002 |  |
| Yes | 295/861 | 1 (Ref.) | 0.98 (0.86-1.12) | 0.93 (0.61-1.41) | 0.96 (0.61-1.51) | 0.772 |  |
| Aspirin use |  |  |  |  |  |  | 0.358 |
| No | 4,692/13,959 | 1 (Ref.) | 1.05 (1.01-1.08) | 0.95 (0.85-1.06) | 1.08 (0.96-1.21) | 0.005 |  |
| Yes | 103/249 | 1 (Ref.) | 0.90 (0.69-1.16) | 0.82 (0.36-1.87) | 0.86 (0.35-2.12) | 0.410 |  |
| Total energy intake |  |  |  |  |  |  | 0.773 |
| <Median | 2,011/7,105 | 1 (Ref.) | 1.14 (0.98-1.34) | 1.34 (1.16-1.54) | 1.10 (0.95-1.28) | 0.018 |  |
| ≥Median | 2,786/7,106 | 1 (Ref.) | 1.05 (0.90-1.22) | 1.08 (0.94-1.24) | 1.11 (0.97-1.27) | 0.136 |  |
| Healthy diets core |  |  |  |  |  |  | 0.079 |
| <Median | 2,258/6,194 | 1 (Ref.) | 0.95 (0.81-1.12) | 1.12 (0.96-1.30) | 1.02 (0.88-1.18) | 0.445 |  |
| ≥Median | 2,539/8,017 | 1 (Ref.) | 1.23 (1.06-1.42) | 1.25 (1.09-1.43) | 1.19 (1.04-1.37) | 0.004 |  |
| **Adenoma** |  |  |  |  |  |  |  |
| Age |  |  |  |  |  |  | 0.697 |
| <60 yr | 919/6,745 | 1 (Ref.) | 1.28 (1.02-1.60) | 1.30 (1.06-1.59) | 1.04 (0.85-1.27) | 0.689 |  |
| ≥60 yr | 1,688/7,466 | 1 (Ref.) | 1.25 (1.06-1.46) | 1.26 (1.09-1.47) | 1.09 (0.93-1.27) | 0.126 |  |
| BMI |  |  |  |  |  |  | 0.517 |
| <24 kg/m2 | 1,415/8,060 | 1 (Ref.) | 1.28 (1.07-1.52) | 1.42 (1.21-1.66) | 1.07 (0.91-1.26) | 0.107 |  |
| ≥24 kg/m2 | 1,192/6,151 | 1 (Ref.) | 1.20 (0.99-1.46) | 1.11 (0.92-1.33) | 1.05 (0.87-1.25) | 0.763 |  |
| Sex |  |  |  |  |  |  | 0.670 |
| Men | 1,770/7,396 | 1 (Ref.) | 1.15 (0.98-1.36) | 1.25 (1.08-1.45) | 1.01 (0.87-1.18) | 0.591 |  |
| Women | 837/6,815 | 1 (Ref.) | 1.40 (1.14-1.72) | 1.29 (1.06-1.58) | 1.17 (0.95-1.44) | 0.079 |  |
| Physical activity |  |  |  |  |  |  | 0.089 |
| <Median | 1,434/7,112 | 1 (Ref.) | 1.27 (1.06-1.51) | 1.37 (1.17-1.61) | 1.14 (0.97-1.35) | 0.031 |  |
| ≥Median | 1,173/7,099 | 1 (Ref.) | 1.25 (1.02-1.52) | 1.18 (0.99-1.42) | 1.00 (0.84-1.20) | 0.981 |  |
| Smoking |  |  |  |  |  |  | 0.201 |
| Nonsmoker | 1,284/9,080 | 1 (Ref.) | 1.26 (1.06-1.51) | 1.35 (1.15-1.58) | 1.15 (0.98-1.36) | 0.023 |  |
| Former/currentsmoker | 1,323/5,131 | 1 (Ref.) | 1.22 (1.01-1.48) | 1.18 (0.99-1.42) | 0.97 (0.81-1.16) | 0.692 |  |
| Alcohol consumption |  |  |  |  |  |  | 0.105 |
| Nondrinker | 1,269/8,174 | 1 (Ref.) | 1.33 (1.13-1.58) | 1.25 (1.06-1.47) | 1.20 (1.01-1.42) | 0.018 |  |
| Drinker | 1,338/6,037 | 1 (Ref.) | 1.12 (0.92-1.38) | 1.26 (1.06-1.51) | 0.97 (0.81-1.15) | 0.811 |  |
| Educational level |  |  |  |  |  |  | 0.521 |
| < middle school | 2,234/12,158 | 1 (Ref.) | 1.27 (1.10-1.46) | 1.25 (1.10-1.42) | 1.07 (0.94-1.21) | 0.220 |  |
| ≥ middle school | 373/2,053 | 1 (Ref.) | 1.17 (0.82-1.68) | 1.36 (0.99-1.88) | 1.12 (0.80-1.56) | 0.347 |  |
| Vitamin consumption |  |  |  |  |  |  | 0.700 |
| <Median | 2,577/14,008 | 1 (Ref.) | 1.03 (0.99-1.07) | 0.94 (0.82-1.08) | 1.05 (0.92-1.21) | 0.149 |  |
| ≥Median | 30/203 | 1 (Ref.) | 1.21 (0.77-1.91) | 0.38 (0.07-2.03) | 1.68 (0.42-6.75) | 0.410 |  |
| Family coloncancer history |  |  |  |  |  |  | 0.207 |
| No | 2,396/12,946 | 1 (Ref.) | 1.03 (0.99-1.08) | 0.94 (0.81-1.08) | 1.06 (0.92-1.23) | 0.108 |  |
| Yes | 152/861 | 1 (Ref.) | 1.01 (0.86-1.18) | 0.92 (0.54-1.57) | 1.02 (0.58-1.78) | 0.938 |  |
| Aspirin use |  |  |  |  |  |  | 0.144 |
| No | 2,569/13,959 | 1 (Ref.) | 1.03 (1.00-1.07) | 0.96 (0.84-1.10) | 1.07 (0.92-1.23) | 0.089 |  |
| Yes | 37/249 | 1 (Ref.) | 0.77 (0.53-1.13) | 0.09 (0.02-0.53) | 0.54 (0.15-1.92) | 0.186 |  |
| Total energy intake |  |  |  |  |  |  | 0.618 |
| <Median | 1,049/7,105 | 1 (Ref.) | 1.41 (1.16-1.70) | 1.28 (1.07-1.53) | 1.12 (0.93-1.35) | 0.115 |  |
| ≥Median | 1,558/7,106 | 1 (Ref.) | 1.13 (0.94-1.35) | 1.26 (1.07-1.48) | 1.02 (0.87-1.20) | 0.560 |  |
| Healthy diets core |  |  |  |  |  |  | 0.063 |
| <Median | 1,272/6,194 | 1 (Ref.) | 0.98 (0.80-1.18) | 1.16 (0.97-1.38) | 0.94 (0.79-1.12) | 0.875 |  |
| ≥Median | 1,335/8,017 | 1 (Ref.) | 1.54 (1.29-1.83) | 1.36 (1.15-1.60) | 1.18 (0.99-1.40) | 0.035 |  |
| ^a^Q, quartile. Multivariable model adjusted for age, sex, BMI (<18.5, 18.5‒24, 24‒28, >28, in kg/m^2^), smoking (never, past smokers with <25 pack-years or ≥25 pack-years, current smokers with <25 pack-years or ≥25 pack-years), alcohol consumption (never, ≤25 mL for men and ≤15 mL for women, >25 mL for men and >15 mL for women), household annual income (yuan), physical activity (MET-h/wk), vitamin supplement use (yes or no), history of family colorectal cancer (yes or no), regular aspirin use (yes or no), educational level (<middle school or ≥middle school), total energy intake (quartile), healthy diet score (quartile). | | | | | | | |

| **Supplement Table 2.** Sensitivity analyses for the multivariable-adjusted ORs (95% CIs) of colorectal polyp or adenoma prevalence according to spicy food consumption ^a^. | | | | | | |
| --- | --- | --- | --- | --- | --- | --- |
|  | **Cases/n** | Quartiles of spicy food consumption (g·2000 kcal^-1^·d^-1^) | | | | |
|  |  | Q1 | Q2 | Q3 | Q4 | P-trend |
| **Polyp** |  |  |  |  |  |  |
| Excluding tindividuals with extreme energy intake | 4629/13,838 | 1 (Ref.) | 1.09 (0.98-1.22) | 1.20 (1.09-1.33) | 1.10 (1.00-1.22) | 0.012 |
| Excluding tindividuals with extreme BMI | 4791/14,195 | 1 (Ref.) | 1.10 (0.98-1.22) | 1.19 (1.08-1.32) | 1.12 (1.01-1.23) | 0.007 |
| Excluding baseline cancer | 4724/13,988 | 1 (Ref.) | 1.04 (1.01-1.07) | 0.95 (0.85-1.06) | 1.08 (0.96-1.21) | 0.016 |
| Further adjust for calcium supplement use | 4797/14,211 | 1 (Ref.) | 1.10 (0.98-1.22) | 1.20 (1.08-1.32) | 1.12 (1.01-1.23) | 0.007 |
| Further adjust for baseline diabetes | 4797/14,211 | 1 (Ref.) | 1.10 (0.98-1.22) | 1.20 (1.08-1.32) | 1.12 (1.01-1.23) | 0.007 |
| **Adenoma** |  |  |  |  |  |  |
| Excluding tindividuals with extreme energy intake | 2517/13,838 | 1 (Ref.) | 1.23 (1.08-1.41) | 1.27 (1.13-1.44) | 1.07 (0.95-1.21) | 0.110 |
| Excluding tindividuals with extreme BMI | 2603/14,195 | 1 (Ref.) | 1.25 (1.09-1.42) | 1.27 (1.13-1.43) | 1.07 (0.95-1.21) | 0.141 |
| Excluding baseline cancer | 2570/13,988 | 1 (Ref.) | 1.02 (0.98-1.06) | 0.94 (0.82-1.08) | 1.07 (0.93-1.23) | 0.257 |
| Further adjust for calcium supplement use | 2607/14,211 | 1 (Ref.) | 1.24 (1.09-1.41) | 1.27 (1.13-1.43) | 1.07 (0.94-1.20) | 0.148 |
| Further adjust for baseline diabetes | 2607/14,211 | 1 (Ref.) | 1.25 (1.09-1.42) | 1.27 (1.13-1.44) | 1.07 (0.95-1.20) | 0.140 |
| ^a^Q, quartile. Multivariable model adjusted for age, sex, BMI (<18.5, 18.5‒24, 24‒28, >28, in kg/m^2^), smoking (never, past smokers with <25 pack-years or ≥25 pack-years, current smokers with <25 pack-years or ≥25 pack-years), alcohol consumption (never, ≤25 mL for men and ≤15 mL for women, >25 mL for men and >15 mL for women), household annual income (yuan), physical activity (MET-h/wk), vitamin supplement use (yes or no), history of family colorectal cancer (yes or no), regular aspirin use (yes or no), educational level (<middle school or ≥middle school), total energy intake (quartile), healthy diet score (quartile). | | | | | | |
